# Fluorescence Differentiation of ATP-Related Multiple Enzymatic Activities in Synovial Fluid as a Marker of Calcium Pyrophosphate Deposition Disease Using Kyoto Green

**DOI:** 10.3390/molecules25051116

**Published:** 2020-03-02

**Authors:** Nattha Yongwattana, Nutsara Mekjinda, Tulyapruek Tawonsawatruk, Itaru Hamachi, Akio Ojida, Jirarut Wongkongkatep

**Affiliations:** 1 Department of Biotechnology, Faculty of Science, Mahidol University, 272 Rama 6 Road, Bangkok 10400, Thailand; oun.nattha@gmail.com (N.Y.); nutsara.mek@student.mahidol.ac.th (N.M.); 2 Department of Orthopedics, Faculty of Medicine, Ramathibodi Hospital, Mahidol University, 270 Rama 6 Road, Bangkok 10400, Thailand; tulyapruek.tao@mahidol.ac.th; 3 Department of Synthetic Chemistry and Biological Chemistry, Graduate School of Engineering, Kyoto University, Kyoto 615-8510, Japan; ihamachi@sbchem.kyoto-u.ac.jp; 4 Graduate School of Pharmaceutical Sciences, Kyushu University, 3-1-1 Maidashi, Higashi-ku, Fukuoka 812-8582, Japan; ojida@phar.kyushu-u.ac.jp

**Keywords:** CPPD, pseudogout, ENPP1, PPase, apyrase, synovial fluid, fluorescence detection, molecular sensor

## Abstract

Calcium pyrophosphate deposition disease (CPPD) is a crystal induced inflammation in joints, and causes severe pain in elderly people. The accumulation of pyrophosphate (PPi) in synovial fluid (SF) results from several enzymatic reactions, especially the highly activated e-NPPs, which catalyze the conversion of ATP to PPi. This study demonstrates the detection of relative catalytic activity of 3 enzymes—ecto-nucleotide pyrophosphatase/phosphodiesterases (e-NPPs), tissue nonspecific alkaline phosphatase (TNAP), and ecto-nucleoside triphosphate diphosphohydrolases (e-NTPDases)—using a single molecular sensor called Kyoto Green. Kyoto Green exhibits excellent performance in sensing the catalytic activity of the commercial representatives of the e-NPPs, TNAP, and e-NTPDases, which are ENPP1, PPase, and apyrase, respectively, in both single-enzyme and multi-enzyme assays. Analysis of SF enzymes in 19 SF samples from human and swine revealed moderate activity of e-NPPs, high activity of e-NTPDases, and low activity of TNAP. Our newly developed method for analysis of multiple enzymatic activities using Kyoto Green in biological SF will assist improvement in accuracy of the CPPD prognosis/diagnosis, which will minimize unnecessary medical procedures.

## 1. Introduction

Articular cartilage calcification is a phenomenon that causes severe pain and inflammation in the joints of elderly individuals, overlapping broadly with osteoarthritis. Among the calcium salts that cause articular cartilage calcification, calcium pyrophosphate dihydrate (CPP) and calcium phosphate are found in over 20% of population over 60 years of age [1], and in 60% of patients considering joint arthroplasty [2]. The deposition of CPP crystal in joints is referred to as calcium pyrophosphate deposition disease (CPPD), also known as pseudogout until recently [3]. Despite its reported high prevalence, CPPD is still underdiagnosed due to the lack of an effective diagnosis tool [2,3]. Currently, the most accurate indicator of CPPD is the positive birefringence of the CPP crystals in synovial fluid from the affected joints [3]; however, the observation of CPP crystals via polarized light microscope is difficult in practice because these crystals are small and often show weak birefringence. Therefore, a more accurate technique to differentiate CPPD from gout and osteoarthritis is needed to help minimize misdiagnosis and increase the quality of life in elderly patients.

**Figure 1:**
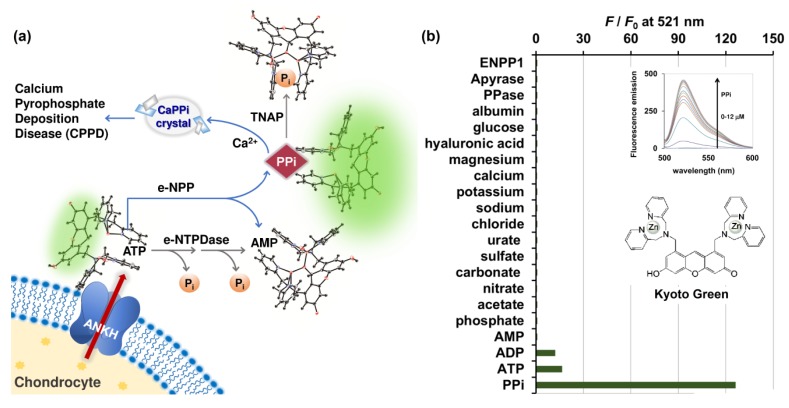
(**a**) Schematic illustration of ATP conversion pathways occurring in biological synovial fluid differentiated using PPi-specific Kyoto Green; (**b**) Selectivity of Kyoto Green (1 μM) towards various ions (2 μM), hyaluronic acid (3 mg/mL), glucose (3 mg/mL), albumin (20 mg/mL), PPase (0.4 ng/μL), apyrase (4 ng/μL), and ENPP1 enzymes (1 ng/μL) commonly found in biological synovial fluids. Measurement conditions: 50 mM HEPES buffer, 10 mM NaCl, 1 mM MgCl_2_ (pH 7.4, 25 °C, *λ*_ex_ = 488 nm); (inset, top) Fluorescence emission change of Kyoto Green upon addition of PPi; (inset, bottom) chemical structure of Kyoto Green.

Inorganic pyrophosphate (PPi) plays a central role in pathogenesis of pseudogout, analogous to the role of urate in gout. The majority of PPi in cartilage is produced from extracellular ATP (eATP), which is effluxed from chondrocyte and regulated by the multipass membrane protein known as ANKH (Figure 1a) [4,5]. Articular chondrocytes maintain eATP at concentrations between 2 and 4 nM [6]. Extracellular nucleotides are metabolized by a variety of ectoenzymes detected in the plasma membrane, including ecto-nucleotide pyrophosphatase/phosphodiesterases (e-NPPs), tissue nonspecific alkaline phosphatase (TNAP) or inorganic pyrophosphatases (PPases), ecto-nucleoside triphosphate diphosphohydrolases (e-NTPDases), and ecto-5′-nucleotidases (e-5′-NT) [7]. In particular, the e-NPPs family hydrolyzes ATP to AMP, and produces PPi in the process [7]. The released PPi, in conjunction with the high calcium concentration of 2.3 mM in the synovial fluid, can lead to formation of CPP crystals [8]. Derfus et al. (1998) reported that, upon of addition of ATP, matrix vesicles produce abundant CPP crystals [9,10]. However, reduction in the amount of CPP crystals was observed when PPases were present, as the enzymes were capable of hydrolyzing PPi at neutral pH [11].

To date, no e-NTPDases have been reported in articular chondrocytes. However, it was reported that movement of joints could mechanically stimulate ATP efflux and trigger the release of NTPdases and e-5′-NT, both of which successively catalyze dephosphorylation of the oncoming ATP into AMP and Pi [7]. As this pathway competes with e-NPPs for ATP, the abundance of active e-NTPDases in the synovial fluid could be linked to lower PPi accumulation, and in turn less likelihood of CPPD (Figure 1a). On the other hand, high activity of e-NPPs and low activity of PPase will enhance PPi accumulation in synovial fluid. Therefore, the relative activity of e-NPPs, PPase, and eNTPDase in biological fluids could serve as a powerful indicator of the PPi deposition and CPP crystal formation, and ultimately help differentiate CPPD patients from those with gout and osteoarthritis.

Several ATP detection platforms have been developed and employed to measure the activity of ATP-related enzymes [12,13,14,15]. However, these methods were designed to analyze only one enzyme at a time, in highly-defined buffer systems. [16,17,18]. Herein, we propose a novel strategy for detection of multiple enzymatic activities in biological milieu, using only one small fluorescent molecular probe. The sensor employed, referred hereafter as Kyoto Green, consists of dipicolylamine-Zn^II^ as a target binding motif and xanthene as a fluorophore (Figure 1). It has high affinity towards PPi, and to a lesser extent ATP and ADP. Kyoto Green possesses a unique sensing mechanism, in which specific binding to the target triggers reversible cleavage of a covalent bond, leading to a change in optical properties of the sensing domain [19]. Specifically, in the absence of target, the bridging water molecule between the two Zn^2+^ centers attacks the xanthene ring and disrupts its conjugated π-system, in effect inhibiting the fluorescence. Upon chelation of the target by the two Zn centers, however, the coordination geometry is reconfigured, resulting in removal of nucleophilic water from the xanthene ring and restoration of its π-conjugation. First applied as a Turn-ON fluorescent ATP sensor by Ojida and Hamachi et al. [12], Kyoto Green has since been successfully adapted for several bioanalytical applications, including fluorescent assay of hydrolysis pathway of diadenosine tetraphosphate [16], organelle-localized multicolor fluorescence probes for imaging of ATP dynamics in living cells [20], detection of nucleic acid amplification in molecular diagnosis of viral infections [21], detection of pathogenic spore-forming bacteria through the intracellular ATP pool [22], and fluorescent visualization of red blood cell CR1-mediated ATP release [23]. In this work, Kyoto Green was employed for measurement of relative activities of ATP-converting enzymes in synovial fluids, to achieve more accurate differentiation of CPPD, gout, and osteoarthritis.

## 2. Results and Discussion

### 2.1. Selectivity and Sensitivity of Kyoto Green

After efflux from the chondrocyte via the multipass membrane ANKH protein, ATP is hydrolyzed via two pathways: 1) through e-NPP, forming PPi which can be subsequently hydrolyzed to Pi via the activated TNAP; and 2) though e-NTPDase, which converts ATP to ADP and AMP simultaneously (Figure 1a). We began by verifying the fluorescent signal of Kyoto Green toward various molecules that are commonly found in biological fluids. When fluorescence emission was measured at 521 nm, the sensor exhibited the highest response to 2 μM of PPi (125-fold increase), and moderate response toward the same concentration of ATP and ADP (16- and 13-fold enhancement, respectively) (Figure 1b and inset). Kyoto Green displayed negligible fluorescence response toward the same concentration of other anions such as phosphate, acetate, nitrate, carbonate, sulfate, urate, and chloride. Other cations such as sodium, potassium, calcium, and magnesium; and other components found in biological synovial fluid including glucose, hyaluronic acid, albumin, ENPP1, apyrase, and PPase also failed to increase fluorescence emission of Kyoto Green (Figure 1b). McMahon et al. (2008) reported the compositions of SF as sodium (136.1 mM), calcium (1.2 ~ 2.4 mM), bicarbonate (9.7 ~ 15.4 mM), and chloride (107.1 mM), together with a small amount of potassium. In addition, SF has 3 ~ 4 g/L of hyaluronic acid, 0.7~1.1 g/L of glucose, and the total protein content of 17.2 g/L, in which 55–70% represented albumin [8]. As none of these compositions could inhibit or be sensed by Kyoto Green, the sensor will fluoresce intensely only when the PPi-producing e-NPP pathway is highly active, but PPase activity is low. When the inorganic pyrophosphatase (PPase) is highly activated, PPi degradation by PPase yields a non-binding Pi, resulting in the decrease in fluorescence from Kyoto Green. The fluorescence response of Kyoto Green also diminishes when ATP is converted to AMP and Pi via e-NTPDase pathway, as Kyoto Green responds to AMP less strongly than ATP. Thus, the enhancement in *F*/*F*_0_ of Kyoto Green could indicate the accumulation of PPi in synovial fluids and a high risk of CPPD, whereas the decrease in fluorescence would suggest that the non-PPi producing pathway dominates the overall ATP conversion, hence a low risk of CPPD.

### 2.2. Assay of Ecto-Nucleotide Pyrophosphatase/Phosphodiesterases (e-NPP), Tissue Nonspecific Alkaline Phosphatase (TNAP), and Ecto-Nucleoside Triphosphate Diphosphohydrolases

**Figure 2:**
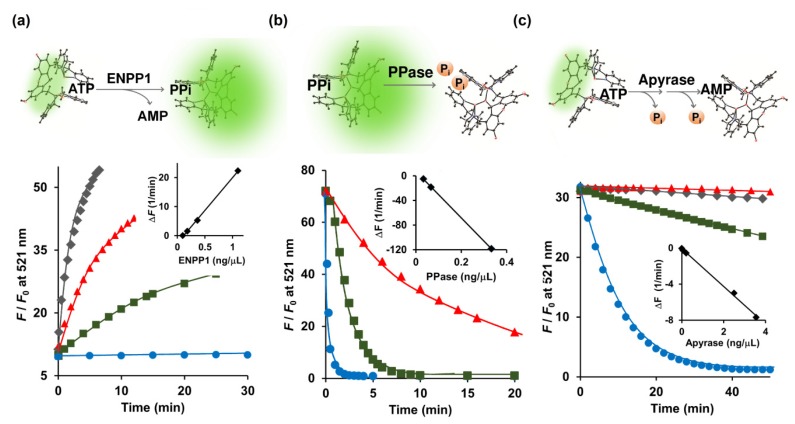
(**a**) Conversion of ATP (5 μM) to PPi catalyzed by ENPP1; (**b**) Degradation of PPi (1 μM) to Pi catalyzed by inorganic PPase; (**c**) Hydrolysis of ATP (2 μM) to AMP catalyzed by apyrase monitored real-time in a 0.7-mL cuvette by 1 μM Kyoto Green. Measurement conditions: 50 mM HEPES buffer, 10 mM NaCl, 1 mM MgCl_2_ (pH 7.4, 25 °C, *λ*_ex_ = 488 nm).

The e-NPPs consist of three established cell surface enzymes: ENPP1, ENPP2, and ENPP3, all of which convert ATP to PPi and AMP [7]. ENPP1, a type-II transmembrane glycoprotein, is located in the extracellular space of mineralizing cells such as osteoblasts and chondrocytes, and assists in the regulation of bone metabolism [24]. Because Kyoto Green prefers PPi over ATP (Figure 1b), conversion of ATP to PPi should increase the fluorescence emission of Kyoto Green. When 5 μM of ATP was hydrolyzed by ENPP1, Kyoto Green yielded approximately 50-fold increase in *F*/*F*_0_ (Figure 2a). On the other hand, when 5 μM ADP was added instead of ATP, the value of *F*/*F*_0_ remained almost constant, yielding Δ*F* of only +0.01 (Table 1), in accordance with the previous finding that showed ADP to be a poor substrate for ENPP1. The initial rates of reaction (i.e., change of fluorescence per min) increased linearly with the amount of ENPP1 added (Figure 2a inset), suggesting that the kinetics of ENPP1 could be studied in one pot with Kyoto Green as a reporter and natural ATP as a substrate. 

Nakagawa et al. (2019) reported the ENPP1 assay based on the Tokyo Green fluorescent dye conjugated with mAMP as a substrate [25]. The conventional ENPP1 assay employs *p*-nitrophenyl 5’-thymidine monophosphate (*p*-Nph-5’-TMP) as a substrate [26,27]. However, Namasivayam et al. (2017) argued that unmodified ATP serves as a better substrate than its derivatized counterpart, *p*-Nph-5’-TMP, because the specificity constant (*k*_cat_/*K*_M_) value of ATP was 6.74-fold higher than that of *p*-Nph-5’-TMP [28]. Furthermore, Lee et al. (2017) also found that *p*-Nph-5’-TMP may also function as an allosteric modulator of ENPP1 by binding to its allosteric site and inducing a conformational change of the active site [29]. Therefore, by directly measuring ATP in its natural form, our Kyoto Green-based ENPP1 assay offers a more accurate way of quantifying ENPP1 activity, free from complications due to chemical derivatization and allosteric modulation.

During calcification of human cartilage, the generated PPi is further hydrolyzed by TNAP to Pi, which is involved in the formation of hydroxyapatite. Lower activity of TNAP will result in the build-up of PPi in the cartilage, posing a higher risk for CPPD. To this end, we sought to investigate whether Kyoto Green can be used to monitor TNAP-catalyzed depletion of PPi in real time. Addition of PPase, a member in TNAP family, to the mixture of PPi and Kyoto Green progressively reduced the fluorescence intensity (Figure 2b). The initial rates of the reaction changed in a linear proportion to the amount of the PPase employed (Figure 2b inset). In contrast, the inorganic PPase exhibited no catalytic activity when ATP and ADP were added as a substrate (Table 1). Taken together, these findings demonstrate that Kyoto Green can serve as an effective indicator in real-time PPase assay. 

Following its efflux from chondrocytes, ATP can be hydrolyzed to ADP and AMP by e-NTPDase. Since Kyoto Green produces moderate fluorescence in response to ATP, e-NTPDase-mediated ATP hydrolysis should lead to reduction in *F*/*F*_0_. Apyrase, a member of the e-NTPDase family, was used as the surrogate for the SF-derived e-NTPDase in this study. Indeed, the addition of apyrase to the ATP-Kyoto Green mixture caused the fluorescence to reduce over time, as shown in Figure 2c. Furthermore, initial reduction rates of *F*/*F*_0_ were in linear relationship with the amount of apyrase added (Figure 2c, inset). Thus, in addition to ENPP1 and PPase, Kyoto Green was also capable of tracking the activity of apyrase.

### 2.3. Relative Activity of ENPP1, Apyrase, and PPase

Several molecular sensors have been developed to monitor catalysis by alkaline phosphatase, PPase, and apyrase in real time [13,30,31,32,33,34]. However, a platform that can detect the activity of more than one enzyme from this group simultaneously is not yet available. Since Kyoto Green had exhibited an excellent response towards each of these enzymes [16], we proceeded to evaluate its performance in a multi-enzyme assay. 

**Figure 3:**
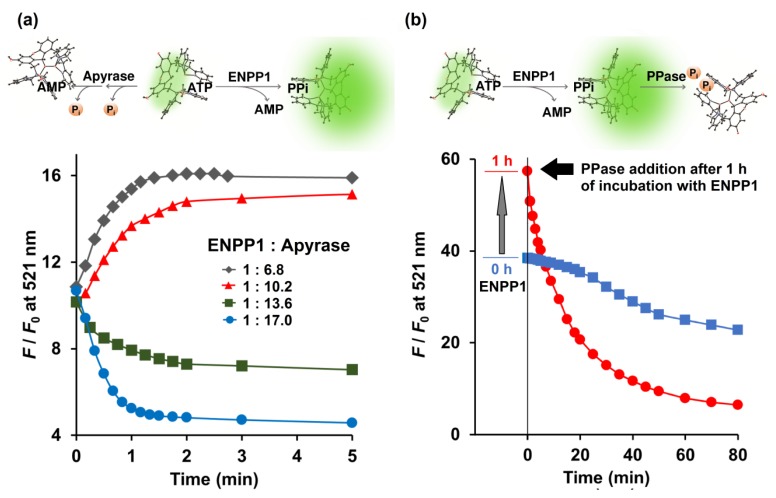
(**a**) Competitive catalytic activity of ENPP1 (0.734 ng/μL) and apyrase (5.0, 7.5, 10.0 and 12.5 ng/μL) toward 5 μM ATP monitored in real time by 1 μM Kyoto Green; (**b**) Catalytic activity of ENPP1 (1.1 ng/μL) when 50 μM of ATP was used as a substrate. PPase (0.03 ng/μL) was added at 0 (blue square) and 1 h (red circle) after ENPP1 addition. Measurement conditions: 50 mM HEPES buffer, 10 mM NaCl, 1 mM MgCl_2_ (pH 7.4, 25 °C, *λ*_ex_ = 488 nm).

Enzymatic conversion of ATP by the combination of ENPP1 and apyrase was monitored in real time by Kyoto Green. Theoretically, active ENPP1 should enhance fluorescence emission at 521 nm due to production of PPi, whereas dephosphorylation of ATP into AMP and Pi by apyrase was expected to reduce the signal. When 0.73 ng/μL ENPP1 was mixed with 5.0 ng/μL of apyrase, overall *F*/*F*_0_ increased over time, indicating that ENPP1-catalyzed PPi production was predominant. Conversely, increasing apyrase concentration to 10.0 ng/μL, while fixing ENPP1 at 0.73 ng/μL, led to suppression of *F*/*F*_0_ (Figure 3a), suggesting that apyrase outperformed ENPP1.

Additionally, Kyoto Green was also used to monitor reactions in which both ENPP1 and PPase were present. A reaction containing 1.1 ng/μL ENPP1 and 50 μM of ATP was allowed to proceed for 1 h, before PPase was added. Upon addition of PPase, *F*/*F*_0_ began to decline sharply (Figure 3b). Decrease of *F*/*F*_0_ was also observed when PPase was present from the beginning, although the change was more gradual, possibly because PPi generated by ENPP1 was immediately consumed by PPase and never allowed to build up (Figure 3b). Collectively, these results demonstrate that, by monitoring real-time changes in fluorescence, Kyoto Green could offer insight on the relative activities of enzymes.

### 2.4. PPi Accumulation Possibility in Biological Fluid

**Figure 4:**
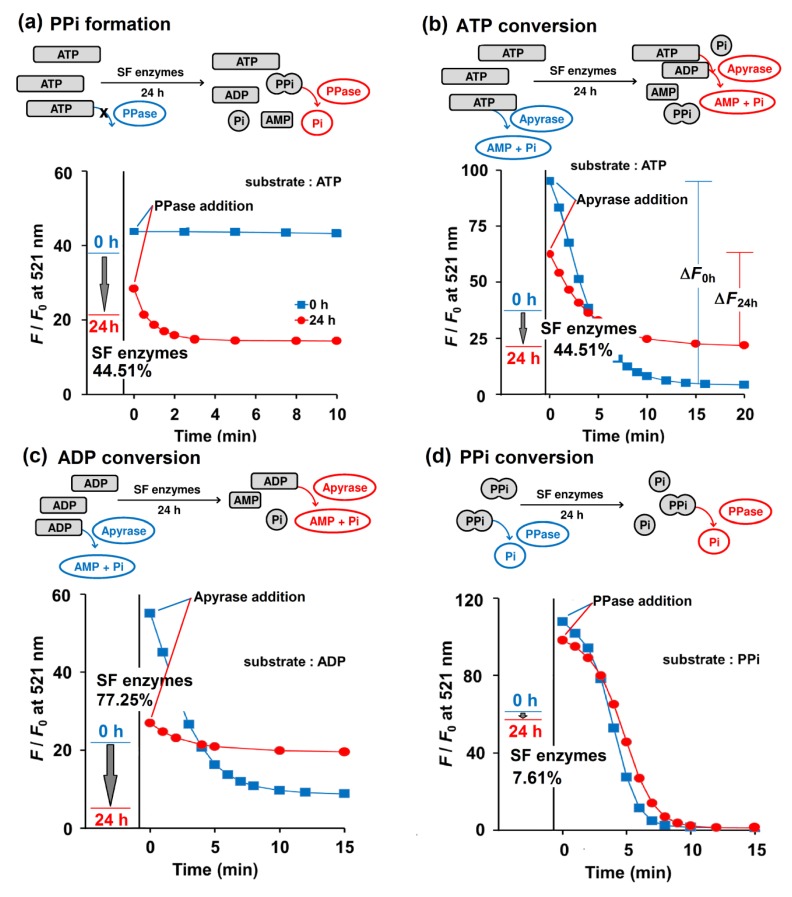
Enzymatic activities of SF No. 1 when 1 mM ATP (**a**, **b**), 1 mM ADP (**c**), and 1 mM PPi (**d**) were employed as a substrate, as reported by Kyoto Green (1 μM). The *F*/*F*_0_ values of Kyoto Green at 0^th^ and 24^th^ h after incubation of the substrate with SF, are displayed as a blue and red line, respectively. The incubated mixtures at 0th (blue square) and 24th h (red circle) were 100-fold diluted and further treated with 0.074 ng/μL PPase (**a**,**d**) and 0.33 ng/μL apyrase (**b**,**c**) then monitored by Kyoto Green (1 μM). Measurement conditions: 50 mM HEPES buffer (pH 7.4, 25 °C, *λ*_ex_ = 488 nm).

To test Kyoto Green’s ability to analyze clinical samples, the sensor was applied to 19 SF samples collected from human and swine as a proof of principle. The SF samples were mixed with the buffer (50 mM HEPES buffer, pH 7.4, containing 1 mM ATP) in the ratio of 1:4 (*v*/*v*) SF: buffer. Each mixture was diluted 1000-fold into the Kyoto Green solution, and their *F*/*F*_0_ values after 0 and 24 h of incubation were recorded (Figure 4). Using the equations as summarized in Table 2, enzymatic activities were then calculated from the *F*/*F*_0_ data (Table 3). The *F*/*F*_0_ ratio of Kyoto Green at 24 h is calculated on the basis of its *F*/*F*_0_ ratio at 0 h and then converted into percentage change. A positive sign in percentage change—i.e., an increase in *F*/*F*_0_ ratio from 0th to 24th h—indicates the predominance of e-NPPs in SF, whereas a negative sign suggests that e-NTPDases dominate. Analysis of SF sample No. 1 revealed a 44.51% decrease of *F*/*F*_0_ from the 0th to 24th h, which suggests that e-NTPDases were the most active enzymes (Figure 4a,b). Percentage changes of *F*/*F*_0_ in samples No. 5, 10 and 11 were slightly positive, indicating that e-NPPs contributed most to the trend. The remaining 16 samples exhibited a large decrease in *F*/*F*_0,_ suggesting that the key metabolic activity in these samples came from e-NTPDases (Table 3).

To validate our interpretations about relative enzymatic activities, reactions were spiked with commercial PPase or apyrase to deplete PPi or ATP, respectively. Addition of PPase to the assay solution of sample No. 1 did not alter *F*/*F*_0_ at 0 h, suggesting that there was no PPi in the solution (Figure 4a). However, after 24 h of incubation, the addition of PPase resulted in a decrease in *F*/*F*_0_ of Kyoto Green over time, indicating that the sample exhibited high propensity for PPi formation (Table 3). The values of PPi formation propensity lower than 5 were interpreted as low risk of PPi formation in SF, symbolized by a minus sign in the Table 2. 

When sample No. 1 was treated with apyrase immediately after mixing with Kyoto Green (i.e., at 0^th^ h of incubation), *F/F_0_* dropped significantly, as indicated by the large Δ*F*_0h_ value. Addition of apyrase after 24 h of incubation reduced *F*/*F*_0_ to a lesser extent (Δ*F*_24h_), which suggests that SF enzymes had partially consumed ATP during the 24 h incubation, leaving less substrate for apyrase (Figure 4b). The percentages of ATP conversion in all samples were calculated according to the equation in Table 2, and the obtained values are summarized in Table 3. We proposed that an ATP conversion of less than 25% should be interpreted as indicating low activity of e-NTPDases, as represented by a negative sign, while a value over 75% would suggest a very high activity (triple positive sign) (Table 2). When the proposed criteria were applied to 19 SF samples investigated, 8 samples were found to exhibit low NTPDases activity, whereas 3 samples had highly active NTPDases (Table 3).

By following similar experimental procedures and calculations as established for ATP, ADP can also be used for assessing overall enzymatic activities in the SF. Table 3 reveals that the ADP conversion by SF enzymes displayed highly negative values, supporting the presence of highly active NTPDases in the SF samples. The results also imply poor efficiency of e-NPPs in converting ADP to PPi. The apyrase assay also shows moderate to high degrees of ADP conversion in 12 out of 19 SF samples, which suggests the presence of active e-NTPDases in the SF. (Table 3).

Similarly, the percentage of PPi conversion could be calculated by comparing the amounts of PPi before and after 24 h of SF enzymatic reaction. PPi abundance at each time point was determined by a PPase assay. In human SF No. 1, the PPi conversion of only 7.61% was observed and the subsequent addition of PPase also yielded comparable Δ*F*_0h_ and Δ*F*_24h_ (Figure 4d). The presence of active TNAP enzymes in the SF is critical because it can reduce the PPi accumulation and in turn the likelihood of CPPD deposition in the joints. Unfortunately, the degree of PPi conversion in 17 of 19 samples was less than 25%. It is also apparent from Table 3 that the PPi formed in Sample No. 1-8 would not have been further degraded to Pi due to the low PPase activity in the SF, which implies a high chance of CPPD. The low activity of PPase in SF of adult and elderly people was also suggested by Stefan et al. (2005) [35].

## 3. Materials and Methods

### 3.1. Materials

Kyoto Green (C_39_H_34_N_6_O_4_Zn_2_•2ClO_4_) was synthesized following Ojida et al [12]. Magnesium chloride (MgCl_2_) was obtained from Merck (Darmstadt, Germany). Sodium chloride (NaCl) was obtained from LobaChemie (Mumbai, India). HEPES (C_8_H_18_N_2_O_4_S), sodium pyrophosphate decahydrate (NaPPi), adenosine, 5’-triphosphate disodium salt (ATP), adenosine 5’-diphosphate disodium (ADP), adenosine 3’-monophosphate (AMP), Apyrase from potatoes was obtained from Sigma-Aldrich Co. (St. Louis, MO, USA). Inorganic pyrophosphatase from yeast (PPase) was purchased from Roche, Rotkreuz, Switzerland. Recombinant Human ENPP1 was purchased from R&D Systems (Minneapolis, MN, USA).

Swine SF samples were obtained from the hind legs of 90–120 kg adult healthy dead pigs from a local slaughter house (Betagro Public Company, Banglen, Nakhon Pathom, Thailand). SF was removed from the knee joint, located 22 cm away from the end of the leg, with a 20 gauge needle at approximately 1 h after death. The samples were stored at −20 °C until further use.

Human SF samples were kindly provided by Asst. Prof. Dr. Tulyapruek Tawonsawatruk, Department of Orthopedics, Faculty of Medicine, Ramathibodi Hospital, Mahidol University, Bangkok, Thailand. These samples were left over from the therapeutic treatment of patients with joint inflammation and display no signs of bacterial infection. The obtained human SF samples were used after ethical approval from Ramathibodi Hospital, Mahidol University, Bangkok, Thailand (Documentary Proof of Ethical Clearance Committee on Human Rights Related to Research Involving Human Subjects, based on the declaration of Helsinki, Faculty of Medicine Ramathibodi Hospital, Mahidol University Reference No. MURA2017/317).

The swine SF and human SF samples were pipetted into Eppendorf tubes and centrifuged at 12,000 rpm (9660 × *g*) for 10 minutes for separation of supernatant and precipitate (WiseSpin CF-10, Daihan Scientific, Gangwon-do, South Korea). The clear supernatant of SF in the upper phase was stored at −20 °C prior to analysis.

### 3.2. Selectivity and Sensitivity of Kyoto Green

The aqueous solution of PPi was serially titrated into a solution (3 mL) of 1 µM Kyoto Green, containing 50 mM HEPES buffer, 10 mM NaCl, 1 mM MgCl_2_, pH 7.4 in a quartz cell at 25 °C. The final concentrations of PPi were between 0.001 and 12 µM. Changes in fluorescence emission from Kyoto Green (1 μM) upon the addition of various anions (2 μM), hyaluronic acid (3 mg/mL), glucose (3 mg/mL), albumin (20 mg/mL), PPase (0.5 ng/μL), apyrase (4 ng/μL), and ENPP1 enzymes (1 ng/μL) were measured with a spectrofluorometer (JASCO FP6500, Tokyo, Japan), using excitation wavelength of 488 nm (25 °C). 

### 3.3. Assay of ENPP1, PPase, and Apyrase

To a solution of Kyoto Green (1 µM) in 50 mM HEPES buffer, 10 mM NaCl, 1 mM MgCl_2_, pH 7.4 placed in a quartz cell, one of the following substrates was added: ATP (5 μM), ADP (5 μM), or PPi (1 μM). Then, an appropriate concentration of ENPP1, PPase, or apyrase was added to the substrate solution and the fluorescence measurements were performed in real time using a spectrofluorometer (JASCO FP6500, Tokyo, Japan) with excitation wavelength of 488 nm and controlled temperature of 25 °C.

### 3.4. Relative Activity of ENPP1, Apyrase, and PPase

The relative activity of ENPP1 and apyrase was measured by addition of 5 μM ATP to the solution of Kyoto Green (1 µM) in 50 mM HEPES buffer (400 µL) containing 10 mM NaCl, 1 mM MgCl_2_, pH 7.4. The concentration of ENPP1 was fixed at 0.734 ng/μL, while the concentrations of apyrase were varied from 5.0 to 12.5 ng/μL. The fluorescence emission of Kyoto Green was recorded over time using a spectrofluorometer (JASCO FP6500, Tokyo, Japan) with excitation wavelength of 488 nm and controlled temperature of 25 °C. 

The conversion of ATP to PPi by ENPP1, and then from PPi to Pi by PPase, was initiated by addition of 1.1 ng/μL of ENPP1 to the 50 μM ATP buffer solution containing 50 mM HEPES buffer (100 µL), 10 mM NaCl, 1 mM MgCl_2_, pH 7.4. The solution was incubated at 37 °C for 0 and 1 h. Then, the mixture was aliquoted in 20 μL to the Kyoto Green (1 μM) solution containing PPase (0.03 ng/μL) in 50 mM HEPES buffer (3 mL), pH 7.4. Fluorescence emission of the Kyoto Green was measured as a time course at excitation wavelength of 488 nm and controlled temperature of 25 °C.

### 3.5. PPi Accumulation Possibility in Biological Fluid

The reaction mixture (RM) containing 1mM of each substrate (ATP, ADP and PPi) dissolved in 50 mM HEPES buffer (120 μL), 1 mM NaCl, 2 mM MgCl_2_ was mixed with 30 μL of the clear supernatant SF and then incubated at 37 °C for 24 h. The solution (3 μL) was then aliquoted at 0th and 24th h of incubation and added to a solution of Kyoto Green (1 µM) in 50 mM HEPES buffer (3 mL), containing 10 mM NaCl, 1 mM MgCl_2_, to obtain fluorescence change of the RM and Kyoto Green at 0 and 24 h.

To estimate the hydrolysis of each substrate (ATP, ADP, and PPi) at 0^th^ and 24^th^ h of incubation with SF at 37 °C, 30 µL of RM was aliquoted and mixed with Kyoto Green (1 μM) and apyrase (0.33 ng/μL) or PPase (0.074 ng/μL) in 50 mM HEPES buffer (3 mL). The fluorescence changes of Kyoto Green were then recorded over time and the calculations performed according to Table 2. Data summarized in Table 3 was an average ± SD of three individual replicates.

## 4. Conclusions

In summary, we report herein the application of a single fluorescent sensor, Kyoto Green, to real-time monitoring of ENPP1, apyrase, and PPase activity. These enzymes are key players in the ATP metabolic pathway, and their malfunctioning may culminate in an accumulation of PPi and development of CPPD in joints. Our novel methods show excellent performance in evaluation of the level of PPi formation, as well as the degree of conversion of ATP, ADP, and PPi to AMP and Pi in human and swine SF specimens. The analysis reveals that 16% of the SF samples demonstrated high propensity for PPi formation, and thus high risks of CPPD. Additionally, 47% of the samples displayed high rates of ADP dephosphorylation to AMP and Pi, which indicates no risk for CPPD. The conversion of PPi to Pi via TNAP in the SF, however, was comparatively low, as 89% of the samples displayed conversion percentage lower than 25%. Future studies should evaluate the ability of our methods to assay SF samples from actual CPPD patients, confirmed cases of which are very rare due to the high uncertainty associated with CPPD diagnosis.

## Figures and Tables

**Table 1:** Maximum fluorescence change (Δ*F*) of Kyoto Green in real-time monitoring of ENPP1, PPase, and apyrase activities.

Substrate	ENPP1	PPase	Apyrase
ATP	+48.72	−0.84	−30.58
ADP	+0.01	−0.89	−16.29
PPi	product ^1^	−70.18	−0.03

^1^ PPi is the product of ATP hydrolysis catalyzed by ENPP1.

**Table 2:** Equations and criteria used for calculation of SF enzymatic activities.

Enzymatic Reactions	Equations	Criteria	Symbols	Interpretation
ATP→(SF enzymes)	100 × [(*F*/*F*_0_)_24h_/(*F*/*F*_0_)_0h_ − 1]	Percentage change	+	e-NPPs predominance
ADP→(SF enzymes)			-	e-NTPDases predominance
PPi→(SF enzymes)				
PPi formation ^1^	Δ*F*_24h_/Δ*F*_0h_	<5	-	Low PPi formation
		5–10	+	
		11–20	++	
		>20	+++	High PPi formation
ATP ^2^/ADP ^2^/PPi ^1^ conversion	1-[Δ*F*_24h_/Δ*F*_0h_]	<0.25	-	Low activity
		0.25–0.50	+	
		0.50–0.75	++	
		>0.75	+++	High activity

^1^ confirmed by PPase. ^2^ confirmed by apyrase.

**Table 3:** ATP-related enzymatic reactions in synovial fluids. The values listed were calculated using the equations summarized in Table 2. The data are arranged from highest to lowest PPi formation.

Substrate		ATP			ADP		PPi	
No.	Source	SF Enzymes	PPi Formation	ATP Conversion	SF Enzymes	ADP Conversion	SF Enzymes	PPi Conversion
1	Human	−44.51 ± 4.25	26.01 ± 1.48 +++	0.53 ± 0.10 ++	−77.25 ± 4.63	0.83 ± 0.13 +++	−7.61 ± 0.21	0.09 ± 0.07 −
2	Swine	−50.22 ± 6.39	23.06±5.96 +++	0.46 ± 0.07 +	−62.89 ± 4.76	0.51 ± 0.18 ++	−11.07 ± 1.47	−0.09 ± 0.05 −
3	Swine	−70.61 ± 0.41	22.68 ± 2.06 +++	0.56 ± 0.02 ++	−84.79 ± 1.25	1.00 ± 0.01 +++	−41.50 ± 9.39	0.19 ± 0.14 −
4	Swine	−59.64 ± 3.82	14.91 ± 1.64 ++	0.52 ± 0.11 ++	−80.82 ± 6.16	0.68 ± 0.18 ++	+36.43 ± 2.21	0.03 ± 0.01 −
5	Swine	+1.27 ± 0.25	12.73 ± 0.19 ++	−0.30 ± 0.17 −	−24.36 ± 2.91	−0.09 ± 0.13 −	+35.12 ± 3.58	−0.11 ± 0.03 −
6	Human	−18.17 ± 0.27	11.20 ± 0.11 ++	0.22 ± 0.03 −	−37.27 ± 2.85	0.31 ± 0.05 +	+8.52 ± 0.07	−0.03 ± 0.00 −
7	Swine	−62.68 ± 5.24	9.07 ± 0.10 +	0.42 ± 0.03 +	−96.10 ± 9.98	1.00 ± 0.17 +++	−43.29 ± 3.57	0.12 ± 0.01 −
8	Swine	−83.83 ± 3.67	5.14 ± 1.02 +	0.75 ± 0.17 +++	−94.64 ± 6.41	1.00 ± 0.09 +++	−62.10 ± 3.52	0.24 ± 0.01 −
9	Swine	−17.2 ± 4.01	4.99 ± 2.39 −	0.19 ± 0.03 −	−47.85 ± 3.50	−0.21 ± 0.07 −	−57.70 ± 7.77	0.23 ± 0.18 −
10	Human	+8.95 ± 7.60	4.95 ± 0.02 −	0.08 ± 0.00 −	−16.36 ± 2.16	0.17 ± 0.01 −	+0.25 ± 0.02	−0.16 ± 0.02 −
11	Swine	+4.46 ± 0.20	3.81 ± 0.16 −	0.14 ± 0.01 −	−47.10 ± 3.75	0.06 ± 0.00 −	−1.56 ± 0.52	0.07 ± 0.01 −
12	Swine	−91.78 ± 8.73	3.58 ± 0.04 −	0.86 ± 0.04 +++	−96.84 ± 6.89	1.00 ± 0.12 +++	−7.14 ± 0.077	0.19 ± 0.01 −
13	Human	−12.15 ± 2.36	2.88 ± 0.05 −	−0.55 ± 0.02 −	−21.44 ± 2.11	−0.21 ± 0.06 −	+7.55 ± 0.96	−0.08 ± 0.02 −
14	Human	−13.85 ± 2.52	1.93 ± 0.01 −	−0.23 ± 0.01 −	−32.06 ± 2.96	−0.13 ± 0.01 −	−3.96 ± 0.17	−0.06 ± 0.01 −
15	Swine	−81.72 ± 6.29	1.00 ± 0.02 −	0.59 ± 0.03 ++	−95.67 ± 8.56	1.00 ± 0.08 +++	−84.74 ± 9.21	0.76 ± 0.06 +++
16	Human	−67.56 ± 2.11	0.55 ± 0.01 −	0.46 ± 0.07 +	−94.44 ± 9.21	1.00 ± 0.11 +++	−7.25 ± 0.058	−0.01 ± 0.00 −
17	Human	−11.36 ± 3.46	0.51 ± 0.01 −	0.09 ± 0.02 −	−35.31 ± 5.47	0.12 ± 0.01 −	−52.72 ± 4.27	0.17 ± 0.01 −
18	Swine	−97.67 ± 4.93	0.46 ± 0.03 −	1.00 ± 0.09 +++	−95.78 ± 6.32	1.00 ± 0.13 +++	−77.79 ± 9.88	0.27 ± 0.02 +
19	Human	−88.51 ± 5.65	0.12 ± 0.00 −	0.74 ± 0.14 ++	−94.06 ± 7.85	0.99 ± 0.11 +++	+4.27 ± 0.46	0.01 ± 0.00 −

## References

[1] Mitrovic D.R., Stankovic A., lriate-Borda O., Uzan M., Quintero M., Miravet L.M., Kuntz D. (1988). The prevalence of chondrocalcinosis in the human knee joint. An autopsy study. J. Rheumatol..

[2] Derfus B.A., Kurian J.B., Butler J.J., Daft L.J., Carrera G.F., Ryan L.M., Rosenthal A.K. (2002). The high prevalence of pathologic calcium crystals in pre-operative knees. J. Rheumatol..

[3] Rosenthal A.K., Ryan L.M. (2016). Calcium Pyrophosphate Deposition Disease. N. Engl. J. Med..

[4] Costello J.C., Rosenthal A.K., Kurup I.V., Masuda I., Medhora M., Ryan L.M. (2011). Parallel regulation of extracellular ATP and inorganic pyrophosphate: Roles of growth factors, transduction modulators, and ANK. Connect. Tissue Res..

[5] Rosenthal A.K., Gohr C.M., Mitton-Fitzgerald E., Lutz M.K., Dubyak G.R., Ryan L.M. (2013). The progressive ankylosis gene product ANK regulates extracellular ATP levels in primary articular chondrocytes. Arthritis Res. Ther..

[6] Graff R.D., Lazarowski E.R., Banes A.J., Lee G.M. (2000). ATP release by mechanically loaded porcine chondrons in pellet culture. Arthritis Rheum..

[7] Graff R.D., Picher M., Lee G.M. (2003). Extracellular nucleotides, cartilage stress, and calcium crystal formation. Curr. Opin. Rheumatol..

[8] Yavorskyy A., Hernandez-Santana A., McCarthy B., McMahon G. (2008). Detection of calcium phosphate crystals in the joint fluid of patients with osteoarthritis–analytical approaches and challenges. Analyst..

[9] Derfus B., Kranendonk S., Camacho N., Mandel N., Kushnaryov V., Lynch K., Ryan L. (1998). Human osteoarthritic cartilage matrix vesicles generate both calcium pyrophosphate dihydrate and apatite *in vitro*. Calcif. Tissue Int..

[10] Derfus A.B., Rachow W.J., Mandel S.N., Boskey L.A., Buday M., Kushnaryov M.V., Ryan M.L. (1992). Articular cartilage vesicles generate calcium pyrophosphate dihydrate like cystals *in vitro*. Arthritis Rheum..

[11] Xu Y., Cruz T.F., Pritzker K.P. (1991). Alkaline phosphatase dissolves calcium pyrophosphate dihydrate crystals. J. Rheumatol..

[12] Ojida A., Takashima I., Kohira T., Nonaka H., Hamachi I. (2008). Turn-on fluorescence sensing of nucleoside polyphosphates using a xanthene-based Zn(II) complex chemosensor. J. Am. Chem. Soc..

[13] Ojida A., Miyahara Y., Wongkongkatep J., Tamaru S.I., Sada K., Hamachi I. (2006). Design of dual-emission chemosensors for ratiometric detection of ATP derivatives. Chem. Asian J..

[14] Jingjing Z., Shizhi Z., Chaoqun N., Chen L., Jie D., Yong C. (2018). A Label-Free Fluorescent DNA Calculator Based on Gold Nanoparticles for Sensitive Detection of ATP. Molecules.

[15] Chaoqun W., Xin Z., Rui L., Zijin Z., Jianyu H., Yi L. (2019). Isotopic core–satellites enable accurate and sensitive bioassay of adenosine triphosphate. Chem. Commun..

[16] Kohira T., Takashima I., Nonaka H., Ojida A., Hamachi I. (2008). Real-time off/on-mode fluorescence assay for enzyme reactions involving nucleoside polyphosphates by use of a xanthene Zn^II^-Dpa chemosensor. Chem. Lett..

[17] Qin L., Wang X., Liu Y., Wei H. (2018). 2D-Metal−organic-framework-nanozyme sensor arrays for probing phosphates and their enzymatic hydrolysis. Anal. Chem..

[18] Huang H., Bai J., Li J., Lei L., Zhang W., Yan S., Li Y. (2019). Fluorometric and colorimetric analysis of alkaline phosphatase activity based on a nucleotide coordinated copper ion mimicking polyphenol oxidase. J. Mater. Chem. B..

[19] Wongkongkatep J., Ojida A., Hamachi I. (2017). Fluorescence sensing of inorganic phosphate and pyrophosphate using small molecular sensors and their applications. Top. Curr. Chem..

[20] Kurishita Y., Kohira T., Ojida A., Hamachi I. (2012). Organelle-localizable fluorescent chemosensors for site-specific multicolor imaging of nucleoside polyphosphate dynamics in living cells. J. Am. Chem. Soc..

[21] Kittiloespaisan E., Ojida A., Hamachi I., Seetang-Nun Y., Kiatpathomchai W., Wongkongkatep J. (2012). Label-free fluorescent detection of loop-mediated isothermal amplification of nucleic acid using pyrophosphate-selective xanthene-based Zn(II)-coordination chemosensor. Chem. Lett..

[22] Tiposoth P., Khamsakhon S., Ketsub N., Pongtharangkul T., Takashima I., Ojida A., Hamachi I., Wongkongkatep J. (2015). Rapid and quantitative fluorescence detection of pathogenic spore-forming bacteria using a xanthene-Zn(II) complex chemosensor. Sens. Actuators B Chem..

[23] Melhorn M.I., Brodsky A.S., Estanislau J., Khoory J.A., Illigens B., Hamachi I., Kurishita Y., Fraser A.D., Nicholson-Weller A., Dolmatova E. (2013). CR1-mediated ATP release by human red blood cells promotes CR1 clustering and modulates the immune transfer process. J. Biol. Chem..

[24] Zimmermann H., Zebisch M., Strauter N. (2012). Cellular functions and molecular structure of ecto-nucleotidases. Purinergic Signal..

[25] Kawaguchi M., Han X., Hisada T., Nishikawa S., Kano K., Ieda N., Aoki J., Toyama T., Nakagawa H. (2019). Development of an ENPP1 fluorescence probe for inhibitor screening, cellular Imaging, and prognostic assessment of malignant breast cancer. J. Med. Chem..

[26] Rachow J.W., Ryan L.M. (1985). Partial characterization of synovial fluid nucleotide pyrophosphohydrolase. Arthritis Rheum..

[27] Lee S.Y., Levesque S.A., Sevigny J., Muller C.E. (2012). A highly sensitive capillary electrophoresis method using *p*-nitrophenyl 5’-thymidine monophosphate as a substrate for the monitoring of nucleotide pyrophosphatase/phosphodiesterase activities. J. Chromatogr. B Analyt. Technol. Biomed. Life Sci..

[28] Namasivayam V., Lee S.Y., Muller C.E. (2017). The promiscuous ectonucleotidase NPP1: Molecular insights into substrate binding and hydrolysis. Biochim. Biophys. Acta..

[29] Lee S.Y., Sarkar S., Bhattarai S., Namasivayam V., De Jonghe S., Stephan H., Herdewijn P., El-Tayeb A., Muller C.E. (2017). Substrate-Dependence of Competitive Nucleotide Pyrophosphatase/Phosphodiesterase1 (NPP1) Inhibitors. Front. Pharmacol..

[30] Zhang X., Chen X., Liu K., Zhang Y., Gao G., Huang X., Hou S. (2020). Near-infrared ratiometric probe with a self-immolative spacer for rapid and sensitive detection of alkaline phosphatase activity and imaging in vivo. Anal. Chim. Acta..

[31] Niu X., Ye K., Wang L., Lin Y., Du D. (2019). A review on emerging principles and strategies for colorimetric and fluorescent detection of alkaline phosphatase activity. Anal. Chim. Acta..

[32] Li Y., Xie R., Pang X., Zhou Z., Xu H., Gu B., Wu C., Li H., Zhang Y. (2019). Aggregation-induced emission fluorescent probe for monitoring endogenous alkaline phosphatase in living cells. Talanta..

[33] Jiang G., Zhu W., Chen Q., Shi A., Wu Y., Zhang G., Li X., Li Y., Fan X. (2017). A new tetraphenylethylene based AIE sensor with light-up and tunable measuring range for adenosine triphosphate in aqueous solution and in living cells. Analyst..

[34] Ghale G., Nau W.M. (2014). Dynamically analyte-responsive macrocyclic host–fluorophore systems. Acc. Chem. Res..

[35] Stefan C., Jansen S., Bollen M. (2005). NPP-type ectophosphodiseterases: Unity in diversity. Trends Biochem. Sci..

